# Supplementary material for: Non‐canonical autophagy functions of ATG16L1 in epithelial cells limit lethal infection by influenza A virus
Source: EMBO J. 2021 Feb 15;40(6):e105543. doi: 10.15252/embj.2020105543 (PMC7957399; doi:10.15252/embj.2020105543)
Supplement: Supplementary file 1 — Appendix [file EMBJ-40-e105543-s003.pdf]

Supplementary Materials for:

Non canonical functions of ATG16L1 in epithelial cells limit lethal infection by influenza A virus

Yingxue Wang, Parul Sharma, Matthew Jefferson, Weijiao Zhang, Ben Bone, Anja Kipar, David Bitto, Janine L. Coombes, Timothy Pearson, Angela Man, Alex Zhekov, Yongping Bao, Ralph A Tripp, Simon R. Carding, Yohei Yamauchi, Ulrike Mayer, Penny P. Powell, James P. Stewart & Thomas Wileman

Correspondence to: j.p.stewart@liv.ac.uk and t.wileman@uea.ac.uk

This PDF file includes:

Figs. S1 to S6

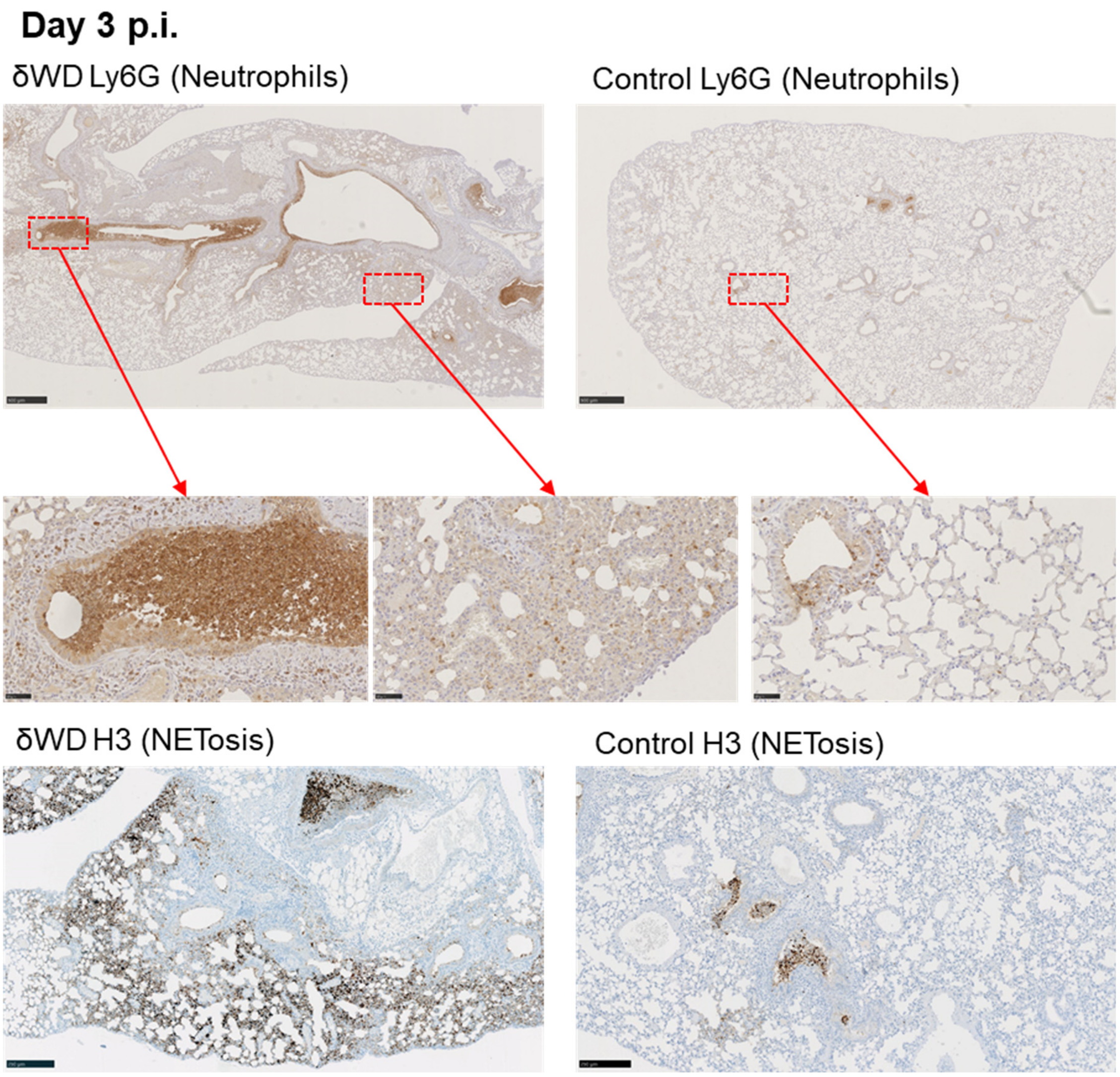

Fig. S1. Increased neutrophilia and NETosis in IAV-infected mice deficient in non-canonical autophagy

δWD and littermate control mice were infected i.n. with 10^3 pfu IAV X31. Lung tissues were harvested at 3d p.i. Neutrophils and H3 (marker of NETosis) were detected by IH using anti-Ly6G and anti-H3, visualized with DAB and counter-stained with hematoxylin. Micrographs of representative areas from lungs of six mice are shown. Scale bars represent 500 µm (upper panels), 50 µm (middle panels) or 250 µm (lower panels). There are dramatically increased numbers of neutrophils in airways (bronchi and bronchioles) and lung parenchyma of δWD mice, accompanied by markedly-increased NETosis, indicating significant neutrophil degeneration.

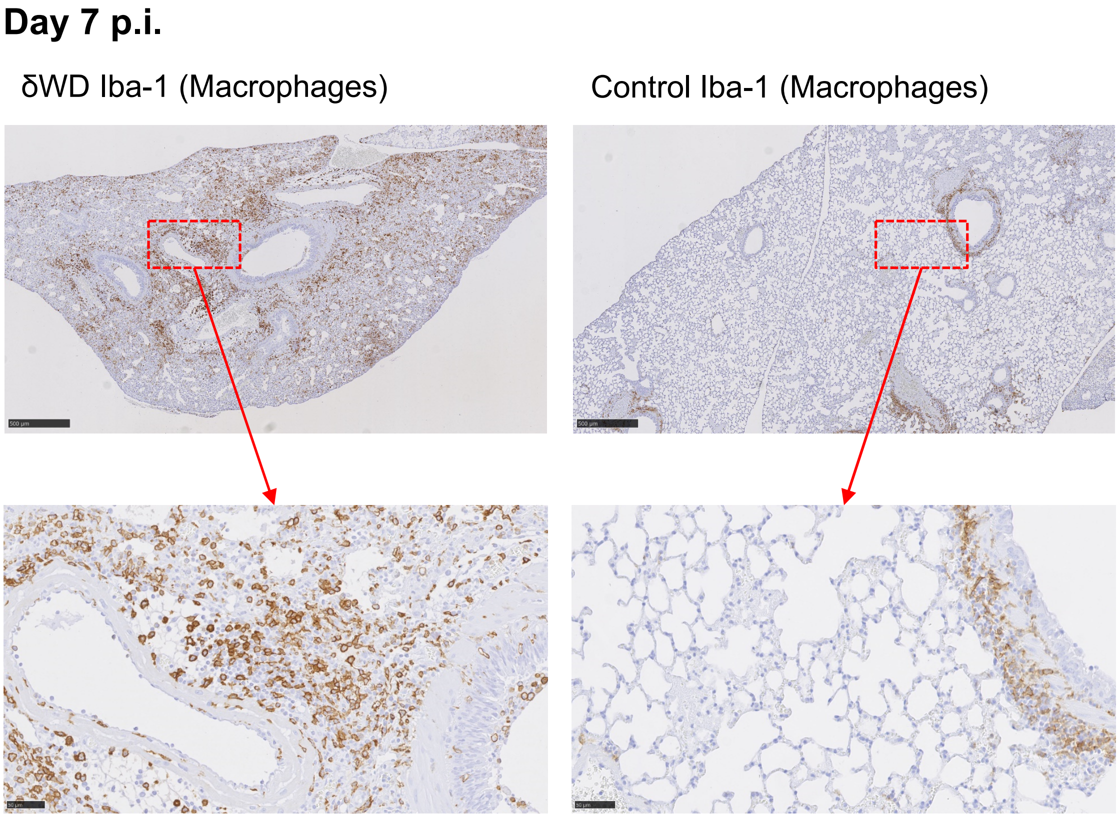

Fig. S2. Increased macrophage rich inflammation in IAV-infected mice deficient in non-canonical autophagy

δWD and littermate control mice were infected i.n. with 10^3 pfu IAV X31. Lung tissues were harvested at 7d p.i. Macrophages were detected by IH using anti-Iba-1, visualized with DAB and counter-stained with hematoxylin. Micrographs of representative areas from lungs of six mice are shown. Scale bars represent 500 µm (upper panels) and 50 µm (lower panels). Lower panels are the same as in Fig. 3B. Upper panels show the lower magnification images of the lung to illustrate the general nature of the observations. There is clearly increased inflammation in δWD mice with higher numbers of macrophages in the lung parenchyma.

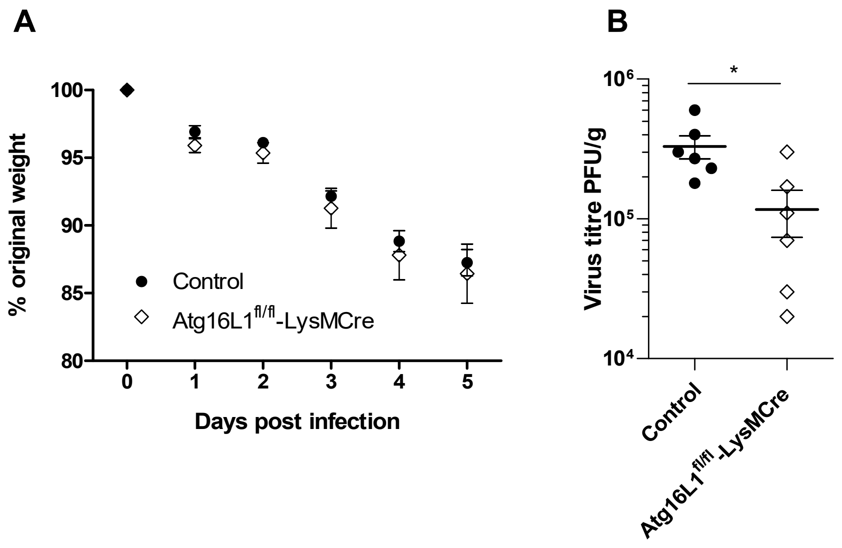

Fig. S3. **LysMcre-mediated loss of canonical macro-autophagy from phagocytes decreases sensitivity to IAV infection**

*Atg*16L1$^{fl/fl}$-LysMcre mice and littermate controls; n = 5 or 6 per group) were infected i.n. with 10^3 pfu IAV X31. **Panel A.** Mice were weighed daily and the weights presented as a percentage of the starting weight. **Panel B.** Lung tissues were taken at 5 d.p.i. and virus titer determined by plaque assay. Data represent the mean value ± SEM. Analysis using the Mann-Whitney U test showed a significant difference (* $p < 0.05$). Thus, *Atg*16L1$^{fl/fl}$-LysMcre mice that are deficient in canonical autophagy in phagocytes lose weight at the same rate as littermate controls but are more resistant to virus replication as they have lower lung virus titres.

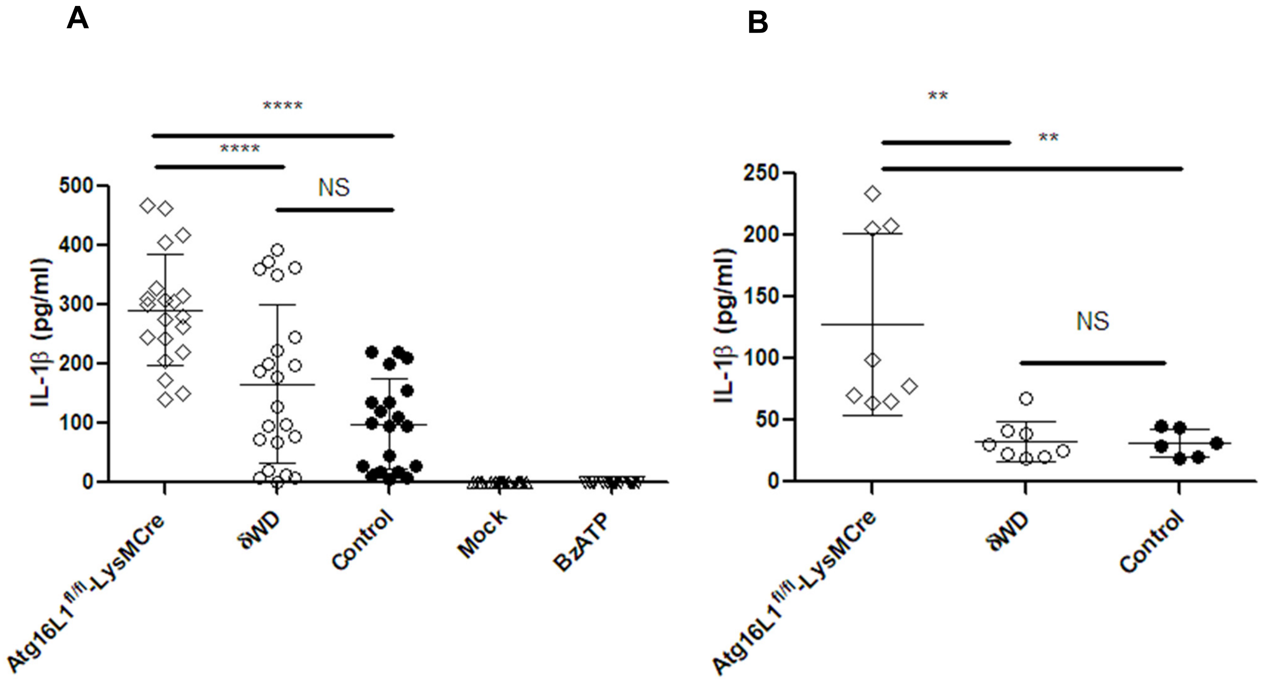

Fig. S4. Mice deficient in non-canonical autophagy do not have elevated IL-1β in response to LPS stimulation

LysMcre-mediated deletion of autophagy genes from mice leads to increased inflammatory threshold characterised by raised secretion of IL-1β from macrophages (*15*), and in the lung this can increase resistance to IAV infection (*16*). The possibility that the δWD mutation could affect IL-1β secretion was tested by stimulating BMDM with bacterial lipopolysaccharide (LPS) and BzATP ($P2X_7$ receptor agonist) or challenging mice with LPS.

Panel A. Bone marrow-derived macrophages (BMDM) from mice strains as indicated were incubated with 100 ng/ml of LPS for 4 h and 150 μM of BzATP for 30 min. Supernatants were assayed for IL-1β by ELISA. (Mock group: untreated, BzATP controls only received BzATP). Representative data are shown as the means ± SD of readings from 20 wells per group and were analyzed using one-way ANOVA with Tukey's post-hoc analysis (**** p<0.0001). Approximately three-fold increases in IL-1β secretion were seen for BMDM from Atg16L1$^{fl/fl}$-LysMCre mice. However, IL-1β secretion from δWD BMDM did not differ significantly from littermate controls.

Panel B. Mouse strains (as indicated) were injected with 20 mg/kg of LPS via the IP route. Serum collected 90 min post injection was assayed for IL-1β by ELISA. In non-treated mice IL-1β was below the detection limit in all 3 strains (not shown). Data are shown as the means ± SD of duplicate assays from 4 mice per group and were analyzed using one-way ANOVA with Tukey's post-hoc analysis (** p < 0.01). Approximately three-fold increases in IL-1β secretion were seen for *Atg*16L1$^{fl/fl}$-LysMCre, however IL-1β secretion for δWD mice did not differ significantly from littermate controls.

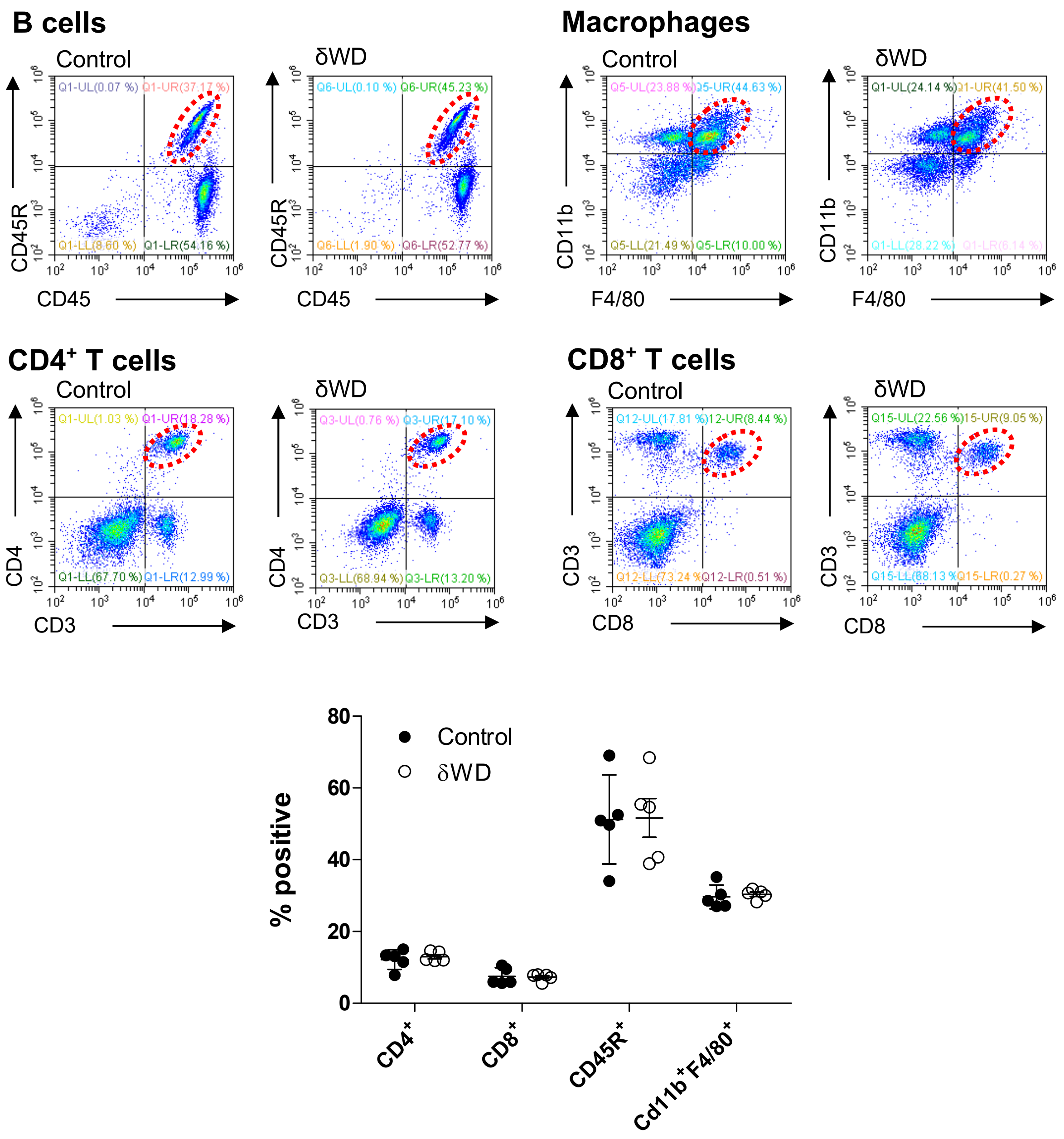

Fig. S5. Mice deficient in non-canonical autophagy have normal leukocyte populations

The possibility that the loss of non-canonical autophagy resulted in changes in leukocyte populations was tested by analysing dissociated spleens by FACS using antibodies to T-cell subsets ($CD3^+$, $CD4^+$ and $CD3^+$, $CD8^+$), B-cells (CD45R/B220) and macrophages (CD11b, F40/80) Upper panel shows representative FACS profiles from n = 3 mice. Lower panel shows the percentage positive for each population.

A

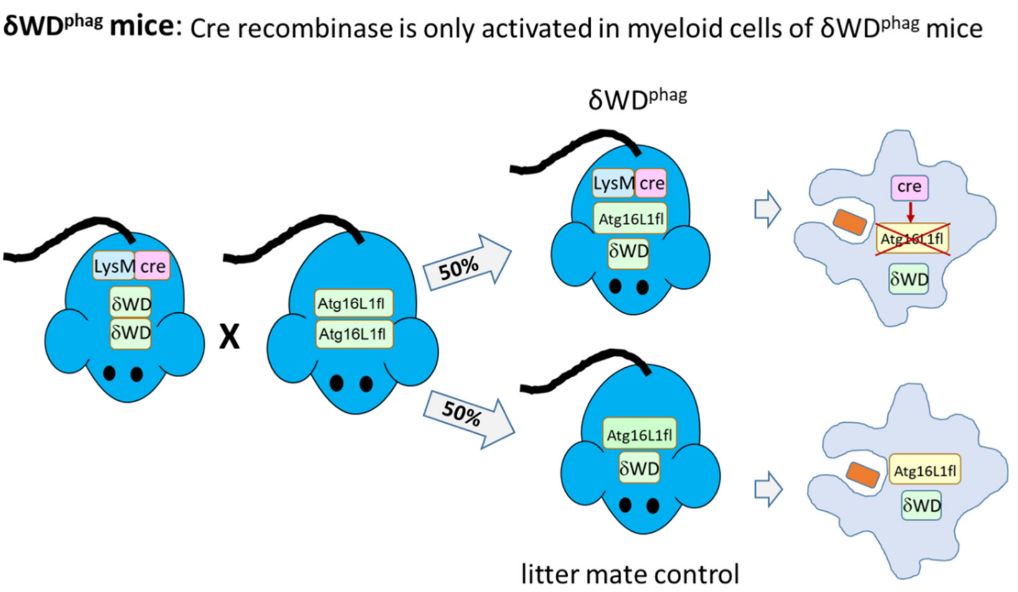
δWDphag mice: Cre recombinase is only activated in myeloid cells of δWDphag mice
δWDphag
LysM cre
δWD
δWD
X
Atg16L1fl
Atg16L1fl
50%
50%
LysM cre
Atg16L1fl
δWD
cre
Atg16L1fl
δWD
Atg16L1fl
δWD
Atg16L1fl
δWD
litter mate control

B

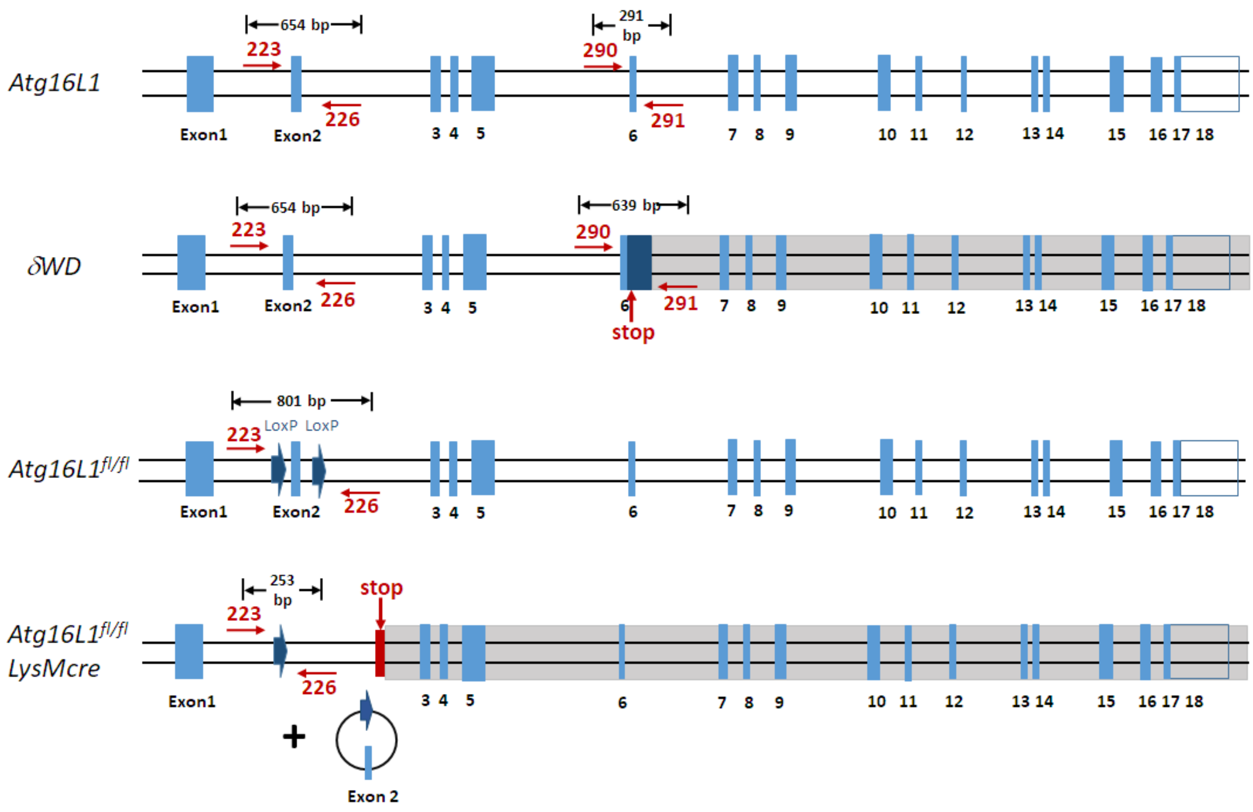
Atg16L1
654 bp
223
226
291 bp
290
291
Exon1
Exon2
3 4 5
6
7 8 9
10 11 12
13 14
15
16 17 18
δWD
654 bp
639 bp
stop
Atg16L1fl/fl
801 bp
LoxP LoxP
Atg16L1fl/fl LysMcre
253 bp
stop
+
Exon 2

C

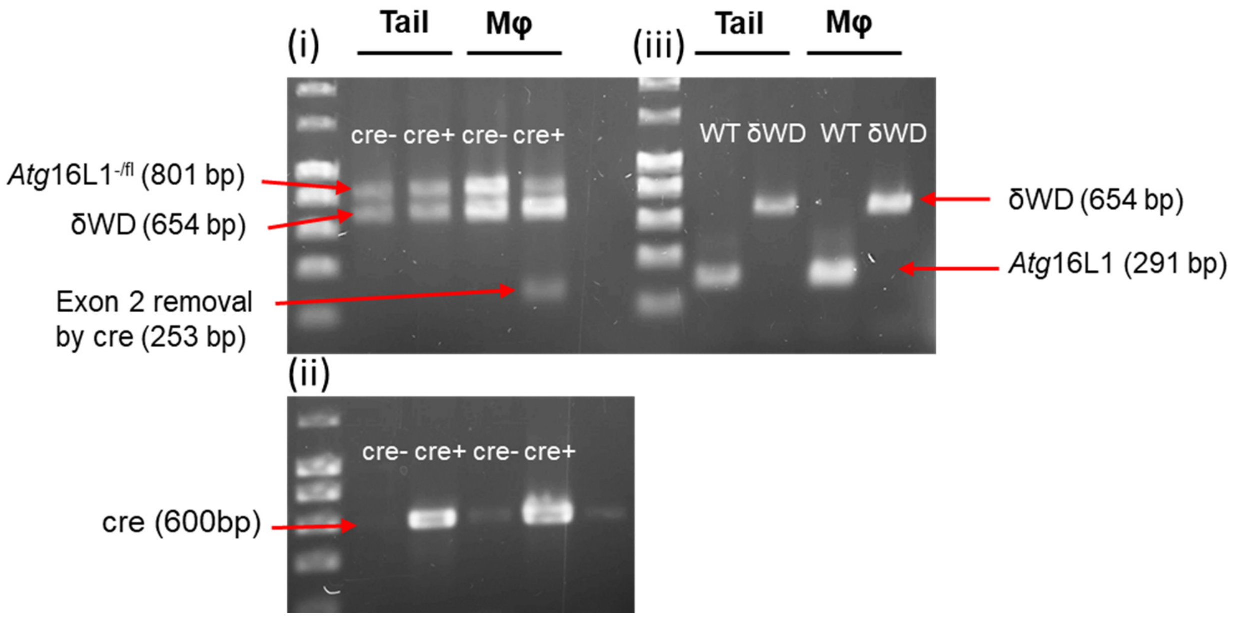

(i)
Tail
Mφ
cre- cre+ cre- cre+
Atg16L1-/fl (801 bp)
δWD (654 bp)
Exon 2 removal
by cre (253 bp)
(iii)
Tail
Mφ
WT δWD WT δWD
δWD (654 bp)
Atg16L1 (291 bp)
(ii)
cre- cre+ cre- cre+
cre (600bp)

D

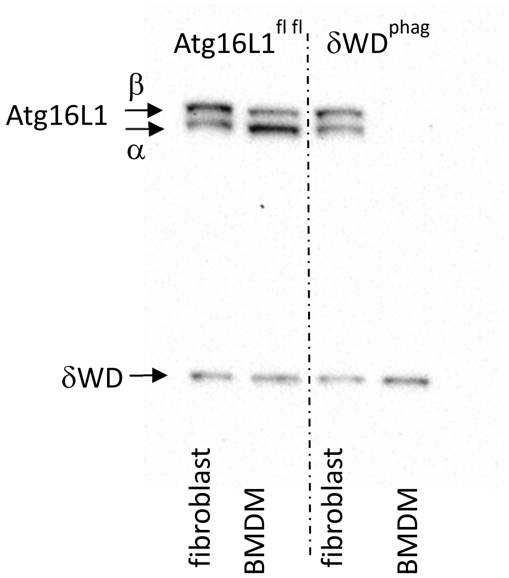

Atg16L1fl fl
δWDphag
Atg16L1
β
α
δWD
fibroblast
BMDM
fibroblast
BMDM

E

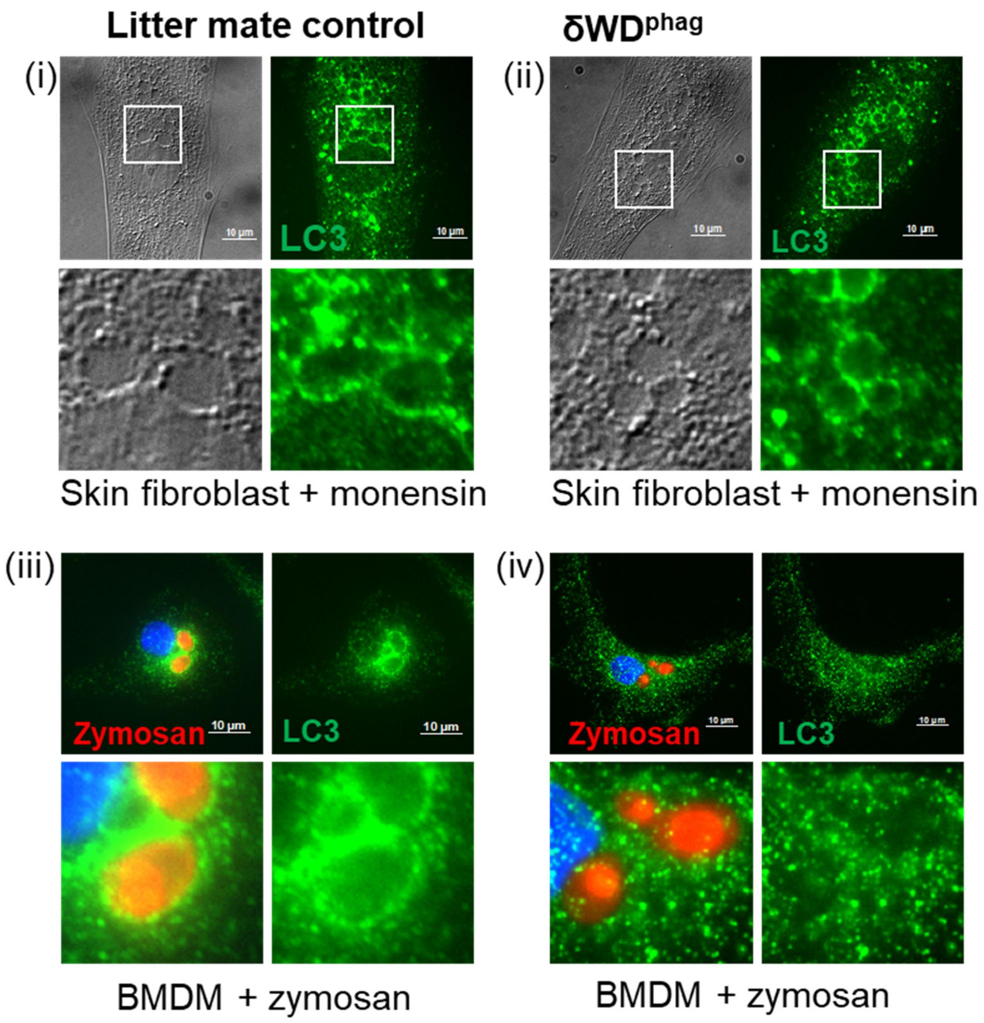

Fig. S6. Generation of δWD^{phag} mice

Panel A. *Breeding strategy.* Homozygous δWD mice carrying LysMcre were crossed with *Atg*16L1$^{fl/fl}$ mice. 50% of progeny are *Atg*16L1$^{fl/δWD}$ and carry LysMcre. Cre recombinase expressed in myeloid cells of these mice inactivates *Atg*16L1 by removing exon 2 from *Atg*16L1 (δWD^{phag}). The myeloid cells only express δWD. Cre recombinase is not expressed in non-myeloid tissues and *Atg*16L1 is preserved to power autophagy. 50% of progeny provide littermate controls because they lack LysMcre and preserve *Atg*16L1 in all tissues.

Panel B. ***Genome map and PCR primers for analysis of the Atg16L1 genotype***. Unmodified *Atg16*L1 is identified using primers flanking exon 2 (223, 226) and exon 6 (290 and 291). The δWD allele was generated by inserting a stop codon into exon 6 and this increases the size of the PCR product of exon 6 from 291 bp to 639 bp. In *Atg*16L1^{fllfl} loxp sites flanking exon 2 in Atg16L1 increase the PCR product of exon 2 from 654 bp to 801 bp, while removal of exon 2 by cre recombinase reduces the PCR product of exon 2 from 801 bp to 253 bp.

Panel C. ***Genotyping δWD^{phag} mice.*** DNA extracted from mouse tail tissue or bone marrow derived macrophages (MΦ) was analysed by PCR. **(i).** Samples from δWD^{phag} mice (indicated by cre+) and littermate controls (cre-). The 253bp PCR product seen in macrophage DNA of cre+ δWD^{phag} strains indicates specific removal of exon 2 from *Atg*16L1 in myeloid cells. **(ii).** PCR primers verify presence of cre recombinase (cre+). iii). Genotyping of wild type and δWD strains showing predicted changes in size of PCR product from exon 6.

Panel D. Tissue specific expression of ATG16L1 and δWD. Skin fibroblasts and bone marrow derived macrophages (BMDM) isolated from *Atg*16L1δWD^{phag} mice (δWD^{phag}) and littermate controls were analysed by western blot. Skin fibrobalasts and BMDM from control mice lacking LysMcre (Atg16L1$^{fl/fl}$) express full length 70kDa α and β isoforms of ATG16L1 and the truncated δWD at 25kDa. δWD^{phag} mice express LysMcre indicated by the removal of full length ATG16L1 from BMDM but not skin fibroblasts.

Panel E. Functional analysis of δWD^{phag} mice

Panels (i) and (ii). Analysis of non-canonical autophagy/LC3 associated endocytosis in fibroblasts from δWD^{phag} mice. Skin fibroblasts isolated from *Atg*16L1δWD^{phag} mice (δWD^{phag}) and litter mate controls were incubated with monensin to induce LC3 associated endocytosis, fixed and immunostained for LC3. Fibroblasts from δWD^{phag} mice are able to recruit LC3 (green) to swollen endo-lysosome compartments in a similar way to those from littermate control mice.

Panels (iii) and (iv). Analysis of non-canonical autophagy/LAP in BMDM from δWD^{phag} mice. BMDMs isolated from *Atg*16L1δWD^{phag} mice (δWD^{phag}) and litter mate controls were incubated with zymosan for 30 min, fixed and immunostained for LC3. BMDMs from δWD^{phag} mice are unable to recruit LC3 (green) to phagosomes containing zymosan (red).
