# Supplementary material for: Non‐canonical autophagy functions of ATG16L1 in epithelial cells limit lethal infection by influenza A virus
Source: EMBO J. 2021 Feb 15;40(6):e105543. doi: 10.15252/embj.2020105543 (PMC7957399; doi:10.15252/embj.2020105543)
Supplement: Supplementary file 2 — Expanded View Figures PDF [file EMBJ-40-e105543-s001.pdf]

Expanded View Figures

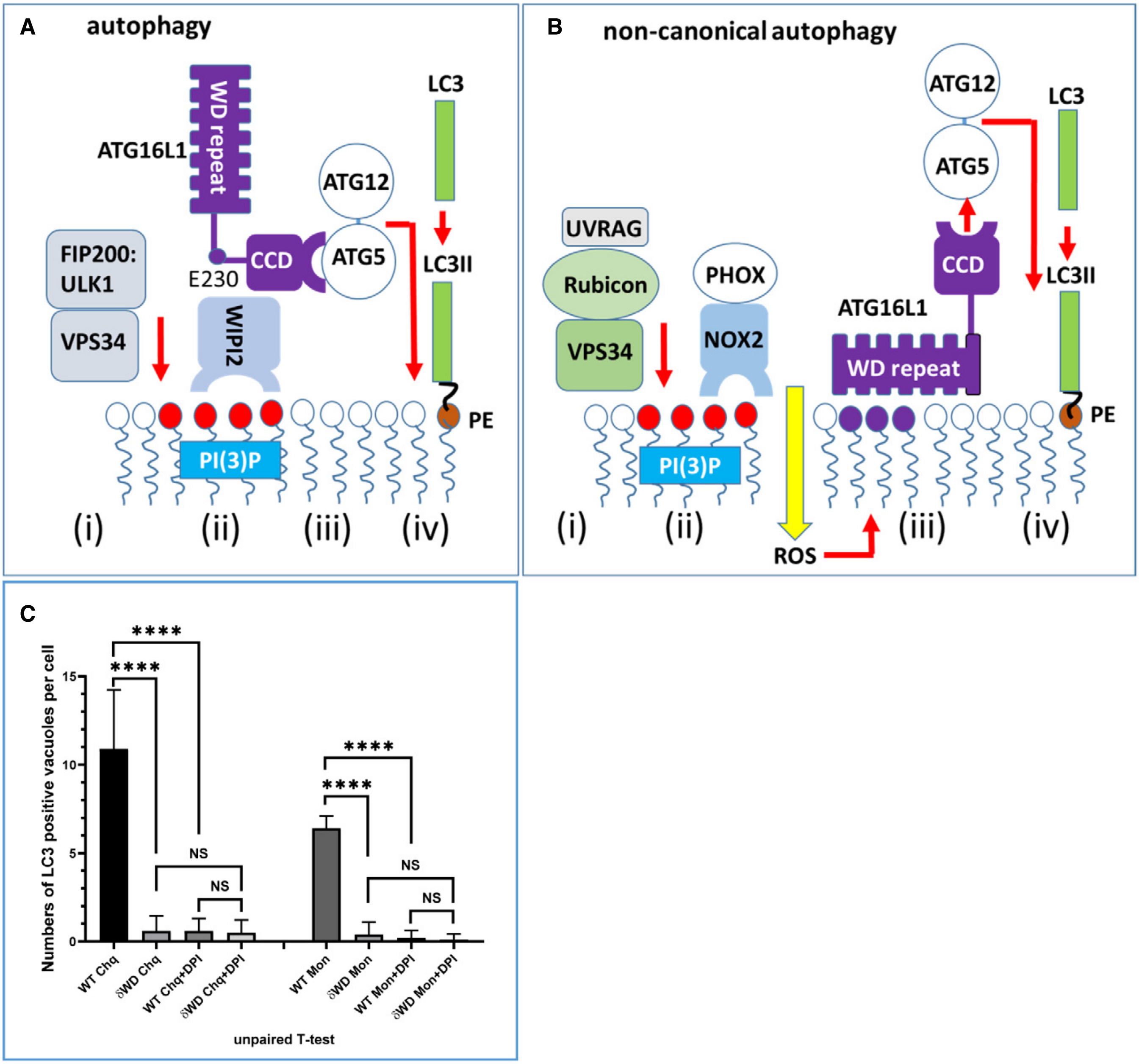

Figure EV1.

Figure EV1. Deletion of the WD domain of ATG16L1 causes selective loss of non-canonical autophagy.

A The WD domain is not required for canonical/conventional autophagy. Conventional autophagy is activated by the initiation complex containing ULK1, FIP200 and the PI3 kinase VPS34 (i) which responds to starvation. VPS34 phosphorylates lipids in membranes to generate sites for WIPI2 binding (ii). WIPI2 binds the coiled coil domain (CCD) and short stretch of linker of ATG16L1 (ii) leading to recruitment of ATG16L1 and the LC3 conjugation machinery (ATG16l1:ATG5-ATG12, ATG7, ATG3) (iii). This results in conjugation of LC3 to PE in the surface of the autophagosome (iv) to promote expansion and fusion with lysosomes.

B The WD domain is required for non-canonical autophagy. During non-canonical autophagy, signalling from the lumen of the endosome or phagosome recruits UVRAG, Rubicon and VPS34 (i). VPS34 phosphorylates lipids in endo-lysosome membranes to generate sites for binding the PHOX:NOX2 complex (ii) which is stabilised by Rubicon to generate reactive oxygen species (ROS). ROS induces binding of the WD domain of ATG16L1 to endo-lysosome membranes (iii) leading to recruitment of the LC3 conjugation machinery (ATG16l1:ATG5-ATG12, ATG7, ATG3) (iii) and conjugation of LC3 to PE (iv).

C ROS is required for recruitment of LC3 to vacuoles in non-phagocytic cells. MEFs from dWD mice or littermate controls were incubated with chloroquine or monensin in the presence or absence of DPI, an inhibitor of NOX2 as indicated. Fixed and permeabilised cells were immunostained for LC3. Recruitment of LC3 to vacuoles was determined by fluorescence microscopy and expressed as numbers of LC3-positive vacuoles per cell ($n = 10$). Recruitment of LC3 required the WD domain of ATG16L1 and was inhibited by DPI. Data from 10 cells are shown, and bars represent the mean ± SD and were compared by Student's *t*-test (*****P* < 0.001).

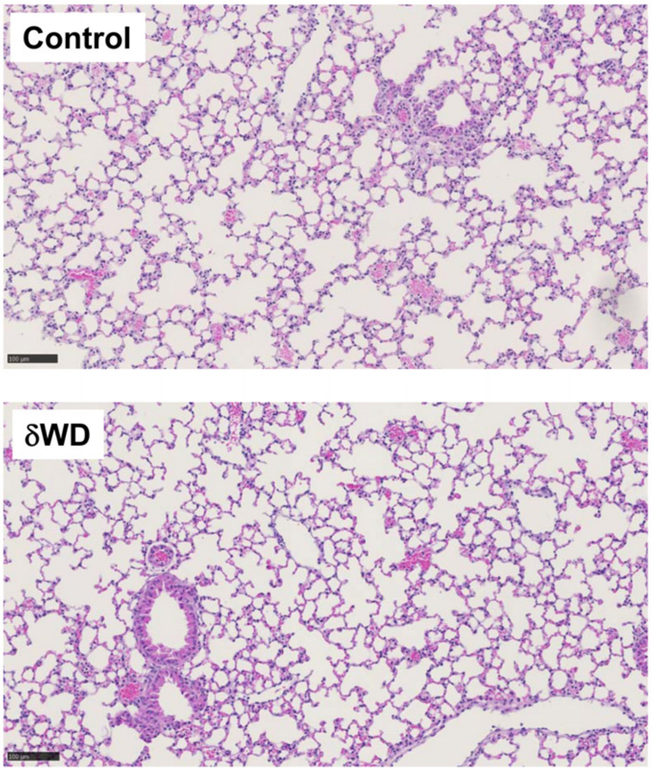

Figure EV2. Histological analysis of littermate control and δWD mice.

Formalin-fixed, paraffin-embedded lung tissue from uninfected littermate control and δWD mice was stained with H&E. Both groups of mice were similar, displaying no histological abnormalities (representative images from $n = 6$). Scale bars represent 100 μM.

 © 2021 The Authors

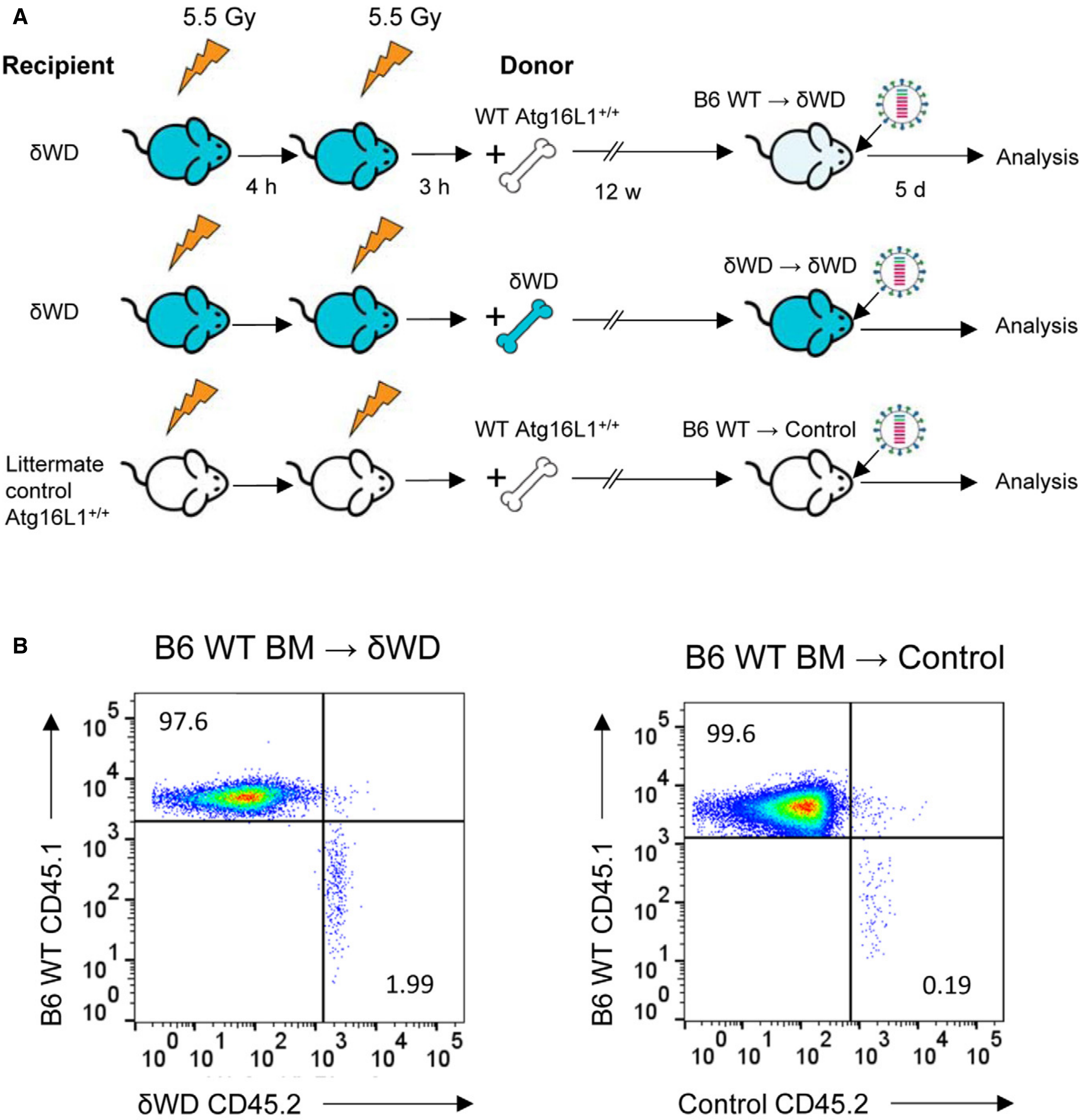

Figure EV3. Confirmation of bone marrow transplant radiation chimaerism.

A Strategy for making bone marrow chimaeras.

B Chimaerism was confirmed 12 weeks post-transplant in spleen cells by flow cytometric analysis of congenic markers on leucocytes (CD45.1, CD45.2). Flow plot shows representative plot from one C57BL/6 WT (CD45.1) bone marrow → δWD (CD45.2) recipient chimaera and one C57BL/6 WT (CD45.1) bone marrow → littermate control (CD45.2) recipient chimaera. All animals were > 95% chimaeric.

© 2021 The Authors

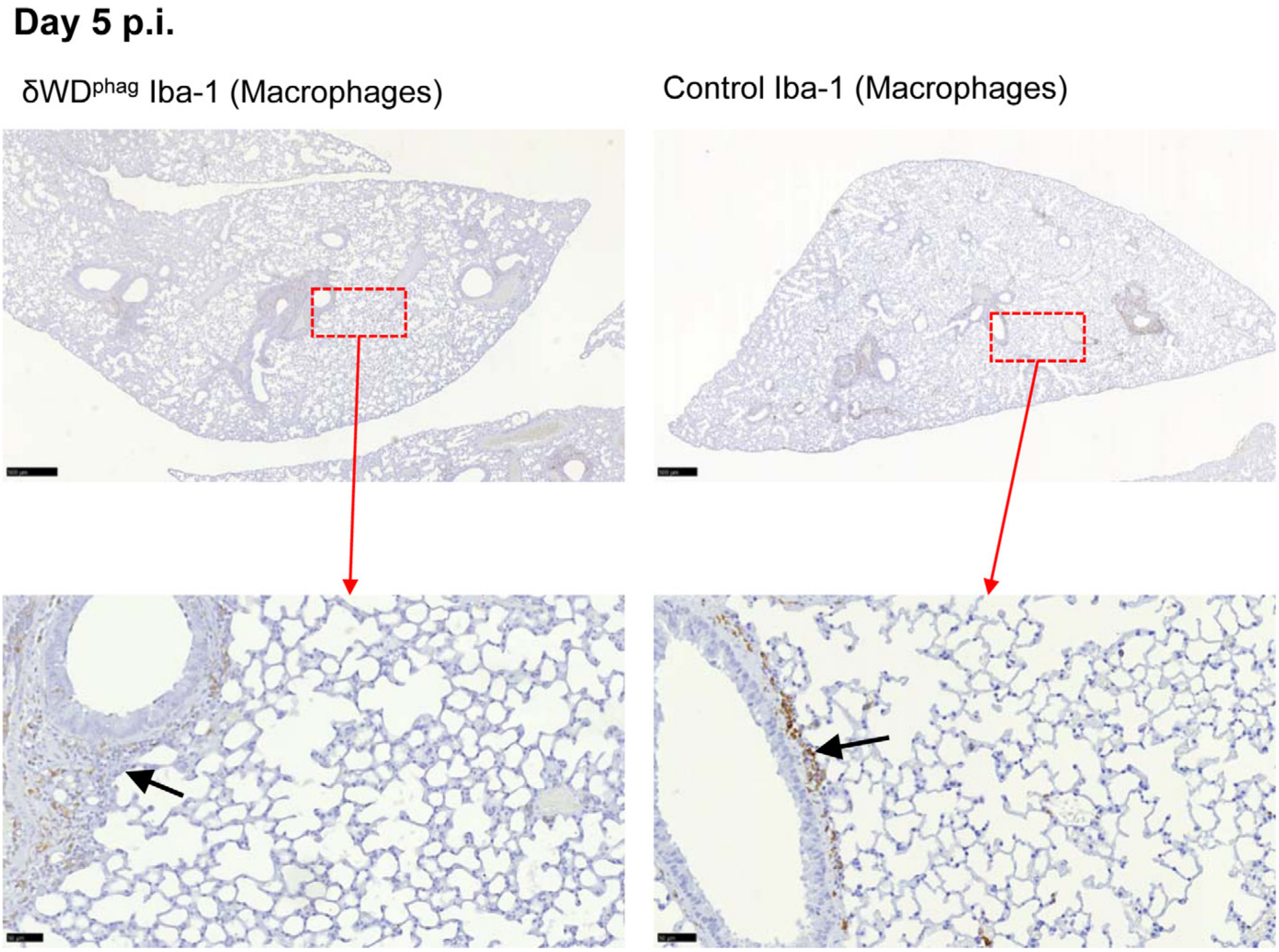

Figure EV4. Mice deficient in non-canonical autophagy/LAP in phagocytes control IAV infection and do not show increased lung inflammation.

δWD^{phag} mice that are deficient in non-canonical autophagy in phagocytes and littermate control mice were infected i.n. with 10^3 pfu IAV X31. Lung tissues were harvested at 5 d p.i. Macrophages were detected by IH using anti-Iba-1, visualised with DAB and counterstained with haematoxylin. Scale bars represent 500 μm (upper panels) and 50 μm (lower panels). Micrographs of representative areas from lungs of six mice are shown. Both δWD^{phag} and control mice show little inflammatory response in the parenchyma and mild, macrophage-rich peri-bronchiolar infiltration (black arrows).

Figure EV5. Non-canonical autophagy reduces endosome fusion *in vitro*.

Sub-confluent monolayers of MEFs were incubated with dual-labelled (SP-DiOC18/R18) IAV at 4°C for 45 min. Cells were harvested by trypsinisation, fixed in PFA and analysed by flow cytometry.

A After labelling, cells were warmed to 37°C for increasing times, harvested by trypsinisation, fixed in PFA and analysed by flow cytometry. Representative plots (from $n = 3$) showing de-quenched SP-DiOC18. Numbers in the gates show percentage of cells positive for fusion as determined by comparison to uninfected cells. Graphical representation of all data is shown in Fig 6E and F.

B–D Fluorescent signal in cells infected with SP-DiOC18-labelled IAV is pH-dependent fusion. pH-dependent fusion was assessed by adding bafilomycin A1 (BAF) to some wells during labelling and then warmed to 37°C for 90 min. (B). Representative plots ($n = 3$) showing de-quenched SP-DiOC18. Numbers in the gates show percentage of cells positive for fusion as determined by comparison to uninfected cells. (C) Percentage of cells positive for fusion. (D) Median fluorescence intensity of de-quenched SP-DiOC18 signal. (C) and (D) show individual replicates ($n = 3$) with a bar at the mean and were compared using Mann–Whitney *U*-test (*$P < 0.05$).

 © 2021 The Authors

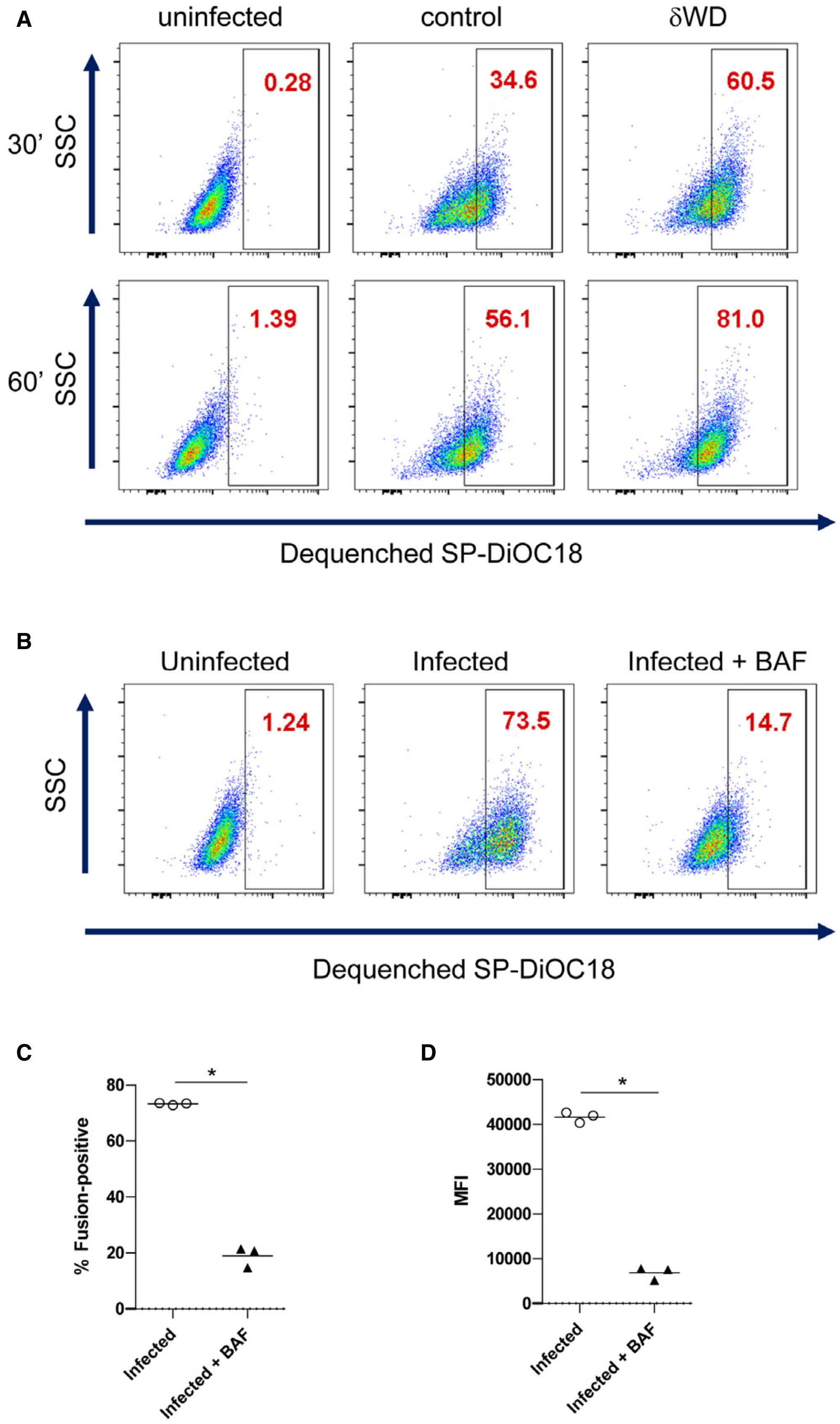
A
uninfected
control
δWD
30'
SSC
0.28
34.6
60.5
60'
SSC
1.39
56.1
81.0
Dequenched SP-DiOC18
B
Uninfected
Infected
Infected + BAF
SSC
1.24
73.5
14.7
Dequenched SP-DiOC18
C
% Fusion-positive
80
60
40
20
0
*
Infected
Infected + BAF
D
MFI
50000
40000
30000
20000
10000
0
*
Infected
Infected + BAF

Figure EV5.
